# Reducing EphA4 before disease onset does not affect survival in a mouse model of Amyotrophic Lateral Sclerosis

**DOI:** 10.1038/s41598-019-50615-0

**Published:** 2019-10-01

**Authors:** Laura Rué, Mieke Timmers, Annette Lenaerts, Silke Smolders, Lindsay Poppe, Antina de Boer, Ludo Van Den Bosch, Philip Van Damme, Wim Robberecht, Robin Lemmens

**Affiliations:** 1 KU Leuven – University of Leuven, Department of Neurosciences, Experimental Neurology and Leuven Brain Institute (LBI), Leuven, Belgium; 2 VIB, Center for Brain & Disease Research, Laboratory of Neurobiology, Leuven, Belgium; 3 University Hospitals Leuven, Campus Gasthuisberg, Department of Neurology, Leuven, Belgium

**Keywords:** Molecular neuroscience, Amyotrophic lateral sclerosis

## Abstract

Amyotrophic lateral sclerosis (ALS) is a neurodegenerative disease that affects motor neurons resulting in severe neurological symptoms. Previous findings of our lab suggested that the axonal guidance tyrosine-kinase receptor EphA4 is an ALS disease-modifying gene. Reduction of EphA4 from developmental stages onwards rescued a motor neuron phenotype in zebrafish, and heterozygous deletion before birth in the SOD1^G93A^ mouse model of ALS resulted in improved survival. Here, we aimed to gain more insights in the cell-specific role of decreasing EphA4 expression in addition to timing and amount of EphA4 reduction. To evaluate the therapeutic potential of lowering EphA4 later in life, we ubiquitously reduced EphA4 levels to 50% in SOD1^G93A^ mice at 60 days of age, which did not modify disease parameters. Even further lowering EphA4 levels ubiquitously or in neurons, did not improve disease onset or survival. These findings suggest that lowering EphA4 as target in ALS may suffer from a complex therapeutic time window. In addition, the complexity of the Eph-ephrin signalling system may also possibly limit the therapeutic potential of such an approach in ALS. We suggest here that a specific EphA4 knockdown in adulthood may have a limited therapeutic potential for ALS.

## Introduction

Amyotrophic lateral sclerosis (ALS) is a neurodegenerative disease that selectively affects motor neurons in the cortex, brainstem and spinal cord, leading to progressive paralysis and eventually to death within 3–5 years after onset^1^. Unfortunately, up to date there are no effective therapies for it. Riluzole and Edaravone, the only Food and Drug Administration-approved drugs for the treatment of ALS, have only a mild effect^2–5^. Since the discovery of genetic mutations in the copper–zinc superoxide dismutase (*Sod1*) gene, other pathogenic mutations in more than 10 different genes have been discovered^6^, being mutations in *Sod1*, chromosome 9 open reading frame 72 (*C9orf72*), fused in sarcoma (*Fus*) and TAR DNA-binding protein 43 (*Tardbp*) the most prevalent. Although it is mainly a mid-life-onset disease, onset and disease duration vary highly among patients^7^. Even patients carrying the same mutation can present with different phenotypic expression of the disease^8,9^. This indicates that there are disease modifying factors, which can be genetic or environmental, that potentially alter disease progression. Getting to know the genetic modifying factors, can lead to the development of new therapies.

We showed previously that one particular tyrosine kinase receptor, the ephrin receptor A4 (EphA4) is an ALS disease-modifying gene. Genetic inhibition of EphA4 rescues the motor neuron phenotype in zebrafish and rodent models of ALS, and EphA4 expression levels inversely correlate with disease onset and survival in patients^10^. EphA4 is a transmembrane receptor involved in short-distance cell-cell communication by interacting with ephrin-A and ephrin-B membrane-anchored ligands^11^. EphA4 is expressed ubiquitously, and within the mouse nervous system its expression can already be detected at E11^12^. Although it is mostly expressed in the hippocampus and in the cerebral cortex, it is also expressed in the brainstem, cerebellum and in the dorsal funiculus and the grey matter of the ventral horn of the spinal cord^12–15^. Interestingly, lack of EphA4 during development can induce dorsal-fated axons of the lumbar spinal cord motor neurons to get a ventral trajectory and innervate ventral muscles of the limb, inducing a defect in hindlimb innervation and posture abnormalities^16^. This suggests that EphA4 has an important function within the spinal cord neuronal population. Interestingly, enhanced gene expression of EphA4 in specific motor neurons correlated with higher vulnerability^10^. Similar beneficial effects of lowering EphA4 levels were observed in mouse models of other neurological diseases, such as stroke, spinal cord injury and multiple sclerosis^17–19^.

However, targeting EphA4 in ALS is complex as shown by recent studies targeting EphA4 after birth in a mouse model of ALS. EphA4-specific antisense oligonucleotides (ASOs) administered directly in the central nervous system (CNS) did not improve disease in the SOD1^G93A^ mouse model of ALS. Motor neuron-specific EphA4 knockdown or the use of an EphA4-blocking recombinant protein administered systemically, had a moderate effect on disease onset in the same model^20,21^. In contrast, the systemic administration of an EphA4-specific agonist improved the survival of SOD1^G93A^ mice^22^. Therefore, there is a need for additional approaches to better understand the modifying role of EphA4 in ALS.

The aim of this study was to gain more insights in the translation potential of reducing EphA4 in ALS, by genetically lowering EphA4 levels from an adult age onwards. In combination with different amount of EphA4 reduction and assessing the cell-specific contribution to disease, our findings suggest that lowering EphA4 expression as target in ALS may suffer from a complex therapeutic time window.

## Results

### EphA4 is efficiently downregulated in mice after Tamoxifen administration

**Figure 1:**
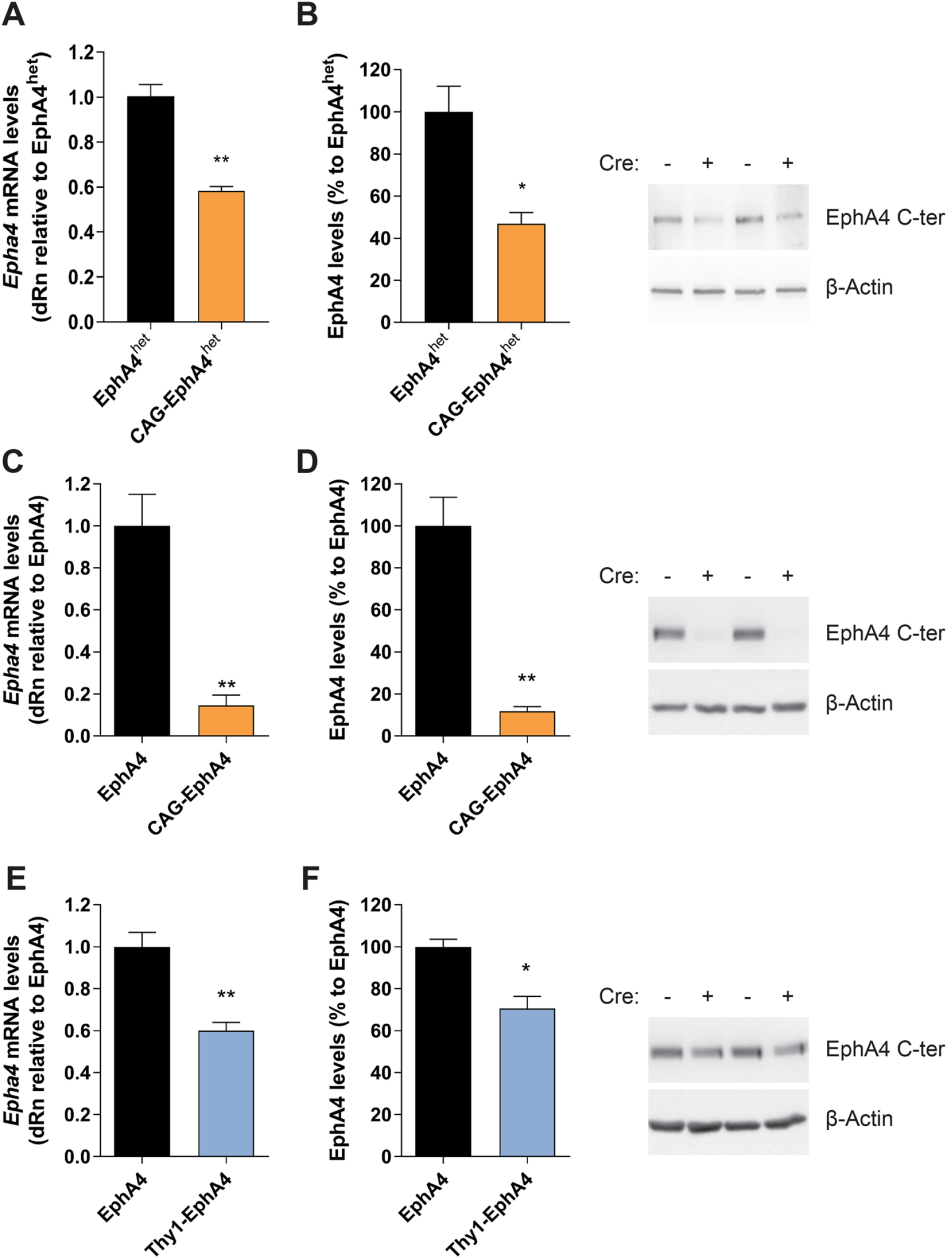
EphA4 mRNA and protein levels are downregulated after Tamoxifen administration. (**A**,**C**,**E**) Gene expression and (**B**,**D**,**F**) protein levels of EphA4 were determined in the lumbar spinal cord of (**A**,**B**) EphA4^F/+^ (EphA4^het^) and CAG-CreER^TM^ EphA4^F/+^ (CAG-EphA4^het^) mice, (**C**,**D**) EphA4^F/F^ (EphA4) and CAG-CreER^TM^ EphA4^F/F^ (CAG-EphA4) mice, and (**E**,**F**) EphA4 and Thy1-CreER^T2^ EphA4^F/F^ (Thy1-EphA4) mice, which all received Tamoxifen 10 days before analysis (N = 3–5). Western blot to detect EphA4 was performed with an EphA4 C-terminal (C-ter) antibody, and β-actin was used as a loading control. Data represents mean ± SEM, and different conditions were compared with a two-tailed t-test: *P < 0.05, **P < 0.01. Representative cropped bands are shown and full-length western blots can be found in Supplementary Material.

EphA4 deletion under the CAG promoter allowed us to reach a ubiquitous reduction of EphA4 mRNA and protein to 50% within the lumbar spinal cord in EphA4^F/+^ CAG-CreER^TM^ (CAG-EphA4^het^) mice, and a loss of more than 85% in EphA4^F/F^ CAG-CreER^TM^ (CAG-EphA4) mice 10 days after Tamoxifen administration as compared with controls (Fig. 1A–D and Supplementary Data, Fig. S1A,B). Neuronal deletion of EphA4 was accomplished in EphA4^F/F^ Thy1-CreER^T2^ (Thy1-EphA4) mice, which showed a 30–40% reduction of EphA4 mRNA and protein levels in the lumbar spinal cord (Fig. 1E,F). This is consistent with the fact that EphA4 is not only expressed in neurons but also in glial cells^18,23^.

**Figure 2:**
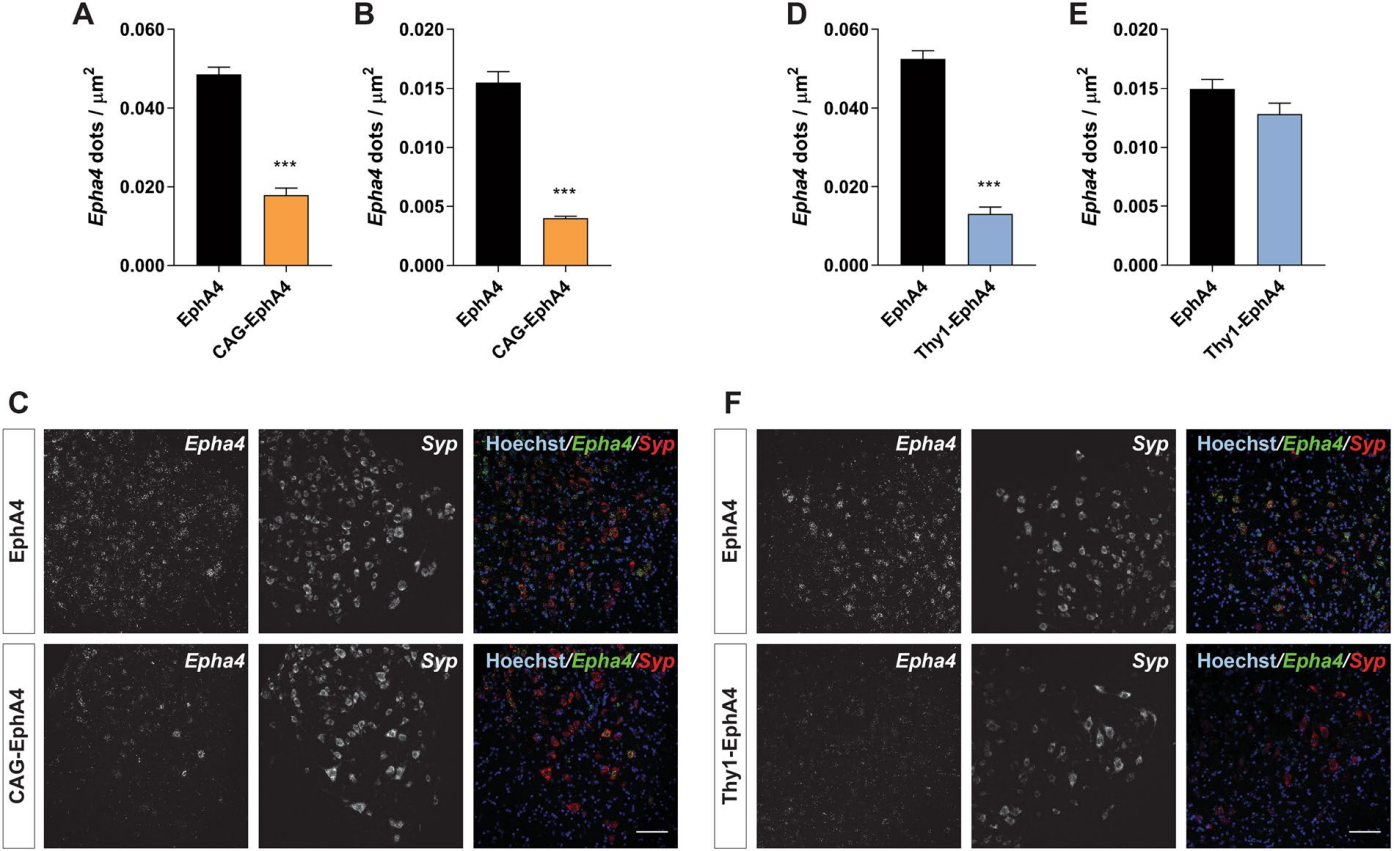
Cell-specific analysis shows ubiquitous reduction of *Epha4* expression in CAG-EphA4 mice and neuronal specific reduction in CAG-Thy1 mice. RNAscope *in situ* hybridization was performed to quantify EphA4 down regulation in neurons and in glial cells in the lumbar spinal cord of (**A**–**C**) CAG-EphA4 mice and in (**D**–**F**) Thy1-EphA4 mice. *Epha4* expression was quantified as the number of *Epha4* dots/µm^2^. Gene expression was quantified in (**A**,**D**) neurons (*Syp* positive) and in (**B**,**E**) glial cells (*Syp* negative). A total of 10–13 images of different ventral horns from 2 to 3 different mice lumbar spinal cords were analysed for every condition. Data represents mean ± SEM, and different conditions were compared with a two-tailed t-test: *** *P* < 0.001. (**C**,**F**) Representative images show the lumbar spinal cord ventral horn of EphA4, CAG-EphA4 and Thy1-EphA4 that were stained with probes against *EphA4* and *Syp*. Hoechst was used as a counter stain for nuclei. Scale bar = 50 µm.

To investigate the cell-type specific EphA4 downregulation, we determined the differential *Epha4* gene expression within neurons and glia at a single-cell level with RNAscope *in situ* hybridization. In CAG-EphA4 mice, *Epha4* expression was reduced in both neurons (*Syp* positive) and glial cells (*Syp* negative; Fig. 2A–C), whereas in Thy1-EphA4 mice, *Epha4* expression was only reduced in the neuronal fraction (Fig. 2D–F).

### Heterozygous deletion of EphA4 levels from 60 days onwards does not slow down disease progression in the SOD1^G93A^ mouse

**Figure 3:**
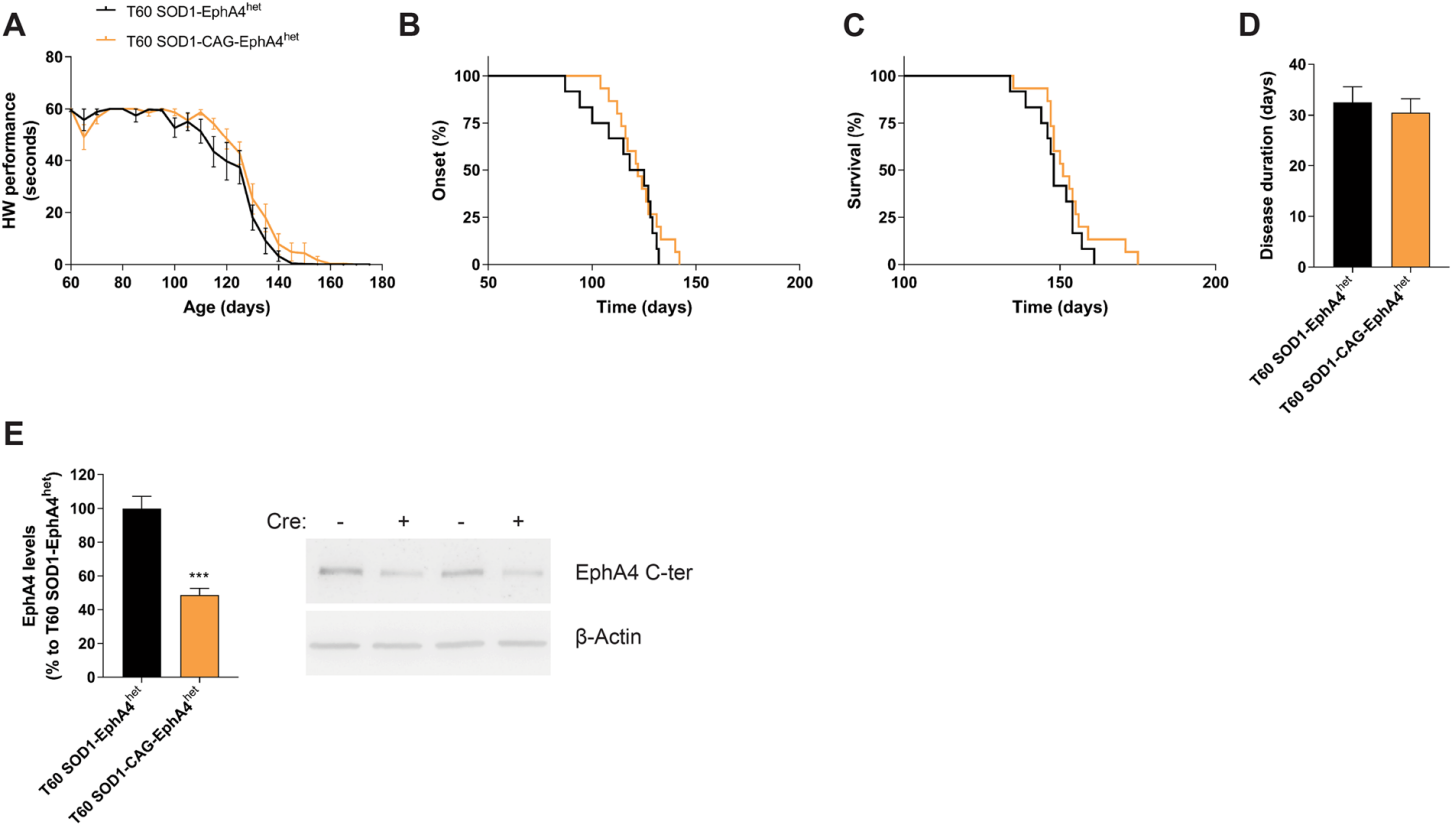
EphA4 deletion to 50% at 60 days of age does not improve disease progression in the SOD1^G93A^ mouse. (**A**–**D**) Disease progression was monitored in SOD1-EphA4^het^ and SOD1-CAG-EphA4^het^ mice, which received Tamoxifen at the age of 60 days (T60). (**A**) Performance decline in the hanging wire test (HW). (**B**) Median disease onset: 121.5 d (SOD1-EphA4^het^, N = 12) and 122 d (SOD1-CAG-EphA4^het^, N = 15), Log-rank p-value = 0.3867. (**C**) Median survival: 148 d (SOD1-EphA4^het^, N = 12) and 151 d (SOD1-CAG-EphA4^het^, N = 15), Log-rank p-value = 0.2892. (**D**) Average disease duration: 32.5 d ± 3.101 d (SOD1-EphA4^het^, N = 12) and 30.53 d ± 2.69 d (SOD1-CAG-EphA4^het^, N = 15), p-value = 0.6348. Two-way ANOVA test with repeated measurements was used to determine differences in the hanging wire performance decline, which is represented as mean ± SEM, whereas the Log-rank test was used for disease onset and survival. Disease progression was analysed with a two-tailed student t-test and represented as mean ± SEM. (**E**) EphA4 protein levels where reduced to about 50% in the lumbar spinal cord end-stage SOD1-CAG-EphA4^het^ mice (N = 7–8) as measured by western blot and compared to EphA4 levels of SOD1-EphA4^het^ mice. Representative cropped bands are shown and full-length western blots can be found in Supplementary Material. EphA4 C-terminal (EphA4 C-ter) antibody was used to quantify EphA4 levels and β-actin was used as a loading control. Data represents mean ± SEM, and different conditions were compared with a two-tailed t-test: ***P < 0.001.

Reducing EphA4 expression with 50% before birth was sufficient to improve survival in a mouse model of ALS^10^. Therefore, we heterozygously decreased EphA4 gene expression at a pre-symptomatic stage of the disease by treating SOD1-CAG-EphA4^het^ and SOD1-EphA4^het^ mice with Tamoxifen at 60 days of age (T60). The motor performance decline assessed on the hanging wire test was not altered in T60 SOD1-CAG-EphA4^het^ mice compared to SOD1-EphA4^het^ mice (Fig. 3A). Disease onset, survival and disease duration were also similar in both experimental groups (Fig. 3B–D). SOD1-CAG-EphA4^het^ mice did not differ from SOD1-EphA4^het^ mice in body weight (Supplementary Data, Fig. S2A). Since we did not observe improvements in disease parameters, the experiment was stopped after including 12–15 mice per group. Reduced EphA4 levels to 50% persisted in SOD1-CAG-EphA4^het^ mice until disease end-stage (Fig. 3E).

### Ubiquitous reduction of EphA4 at day 60 to more than an 85% does not alter disease parameters in the SOD1^G93A^ mouse

**Figure 4:**
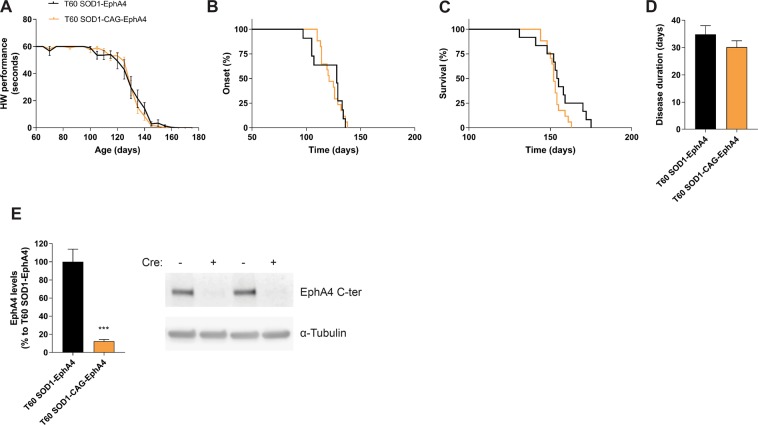
Ubiquitous EphA4 deletion to more than 85% does not improve the phenotype in the SOD1^G93A^ mouse model. Disease progression was closely monitored in (A-D) SOD1-EphA4 and SOD1-CAG-EphA4 mice that received Tamoxifen at the age of 60 days (T60). (**A**) Performance decline in the hanging wire test (HW). (**B**) Median disease onset: 128 d (SOD1-EphA4, N = 11) and 121 d (SOD1-CAG-EphA4, N = 17), Log-rank p-value = 0.9591. (**C**) Median survival: 154.5 d (SOD1-EphA4, N = 12) and 152 d (SOD1-CAG-EphA4, N = 17), Log-rank p-value = 0.1091. (**D**) Average disease duration: 34.82 d ± 3.213 d (SOD1-EphA4, N = 11) and 30.12 d ± 2.387 d (SOD1-CAG-EphA4, N = 17), p-value = 0.2428. Two-way ANOVA test with repeated measurements was used to determine differences in the hanging wire performance decline, which is represented as mean ± SEM, whereas the Log-rank test was used for disease onset and survival. Disease progression was analysed with a two-tailed student t-test and represented as mean ± SEM. (**E**) EphA4 protein levels in the lumbar spinal cord were still decreased in end-stage SOD1-CAG-EphA4 as measured by western blot (N = 4–7). Representative cropped bands are shown and full-length western blots can be found in Supplementary Material. EphA4 C-terminal (EphA4 C-ter) antibody was used to quantify EphA4 levels and α-tubulin was used as a loading control. Data represents mean ± SEM, and different conditions were compared with a two-tailed t-test: ***P < 0.001.

Since 50% deletion of EphA4 did not improve disease parameters, we determined whether a more robust genetic deletion of EphA4 in adult SOD1^G93A^ mice could beneficially modify the disease parameters. We administered Tamoxifen in SOD1-CAG-EphA4 mice and littermate controls, SOD1-EphA4 mice, at the age of 60 days. T60 SOD1-CAG-EphA4 mice and T60 SOD1-EphA4 mice had a similar motor performance decline over time (Fig. 4A), and onset of the disease did not differ in the two groups (Fig. 4B). Both groups reached disease end-stage at the same age, and had a similar disease duration (Fig. 4C,D). The experiment was stopped with 12–17 mice per group since we did not observe improvements in disease parameters. We confirmed a reduction of 88% of EphA4 levels by western blot of lumbar spinal cord tissue from end-stage T60 SOD1-CAG-EphA4 mice compared to T60 SOD1-EphA4 control mice (Fig. 4E). Body weight was not altered in T60 SOD1-CAG-EphA4 mice compared to T60 SOD1-EphA4 mice (Supplementary Data, Fig. S2B).

### Neuronal specific EphA4 deletion does not improve disease in the SOD1^G93A^ mouse

**Figure 5:**
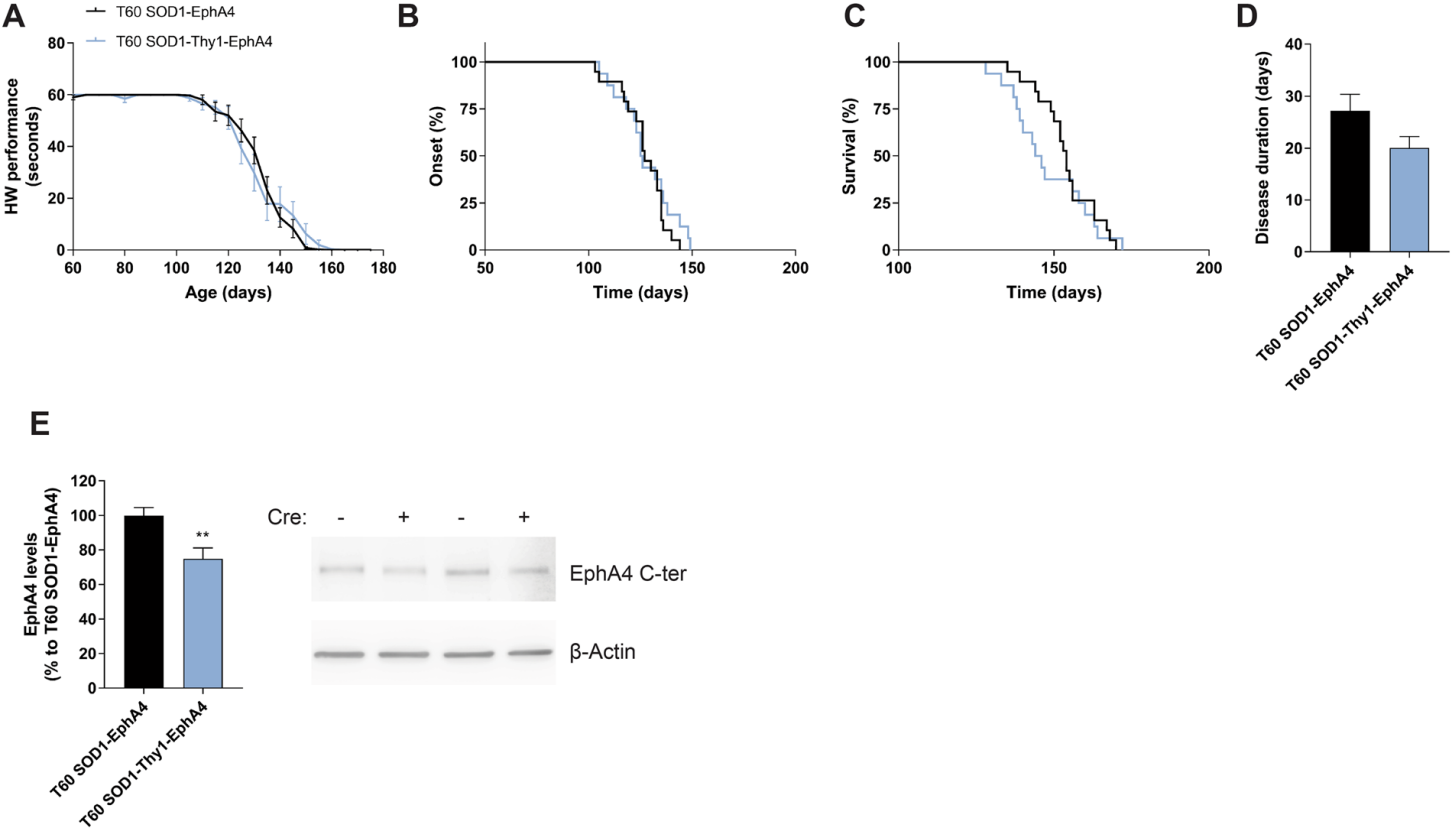
Neuronal EphA4 deletion does not improve the phenotype in the SOD1^G93A^ mouse model. Disease progression was monitored in (**A**–**D**) SOD1-EphA4 and SOD1-Thy1-EphA4 mice that received Tamoxifen at the age of 60 days (T60). (**A**) Performance decline in the hanging wire test (HW). (**B**) Median disease onset: 127 d (SOD1-EphA4, N = 19) and 125.5 d (SOD1-Thy1-EphA4, N = 16), Log-rank p-value = 0.4497. (**C**) Median survival: 154 d (SOD1-EphA4, N = 19) and 145 d (SOD1-Thy1-EphA4, N = 16), Log-rank p-value = 0.493. (**D**) Average disease duration: 27.16 d ± 3.208 d (SOD1-EphA4, N = 19) and 20.06 d ± 2.159 d (SOD1-Thy1-EphA4, N = 16), p-value = 0.087. Two-way ANOVA test with repeated measurements was used to determine differences in the hanging wire performance decline, which is represented as mean ± SEM, whereas the Log-rank test was used for disease onset and survival. Disease progression was analysed with a two-tailed student t-test and represented as mean ± SEM. (**E**) EphA4 protein levels in the lumbar spinal cord were still decreased in end-stage SOD1-Thy1-EphA4 as measured by western blot (N = 3–4). Representative cropped bands are shown and full-length western blots can be found in Supplementary Material. EphA4 C-terminal (EphA4 C-ter) antibody was used to quantify EphA4 levels and β-actin was used as a loading control. Data represents mean ± SEM, and different conditions were compared with a two-tailed t-test: **P < 0.01.

EphA4 gene expression is higher in the most vulnerable spinal cord motor neurons in ALS, and reducing EphA4 levels from developmental stages onwards not only prevents these motor neurons to degenerate in SOD1^G93A^ mice, but also improves axonal regeneration after sciatic nerve axotomy^10^. To determine the neuron-specific contribution of EphA4 in SOD1^G93A^ mice and to assess whether only knocking down the neuronal EphA4 would be of benefit for the disease, SOD1-Thy1-EphA4 mice were treated with Tamoxifen at 60 days of age. Neuronal EphA4 knockdown resulted in similar motor performance as in controls, as assessed with the hanging wire test (Fig. 5A). Disease onset, survival and disease duration did not differ between genotypes (Fig. 5B–D). The lack of improvement in disease parameters was the reason to stop the experiment with 16–19 mice per group. Western blots of lumbar spinal cord tissue from end-stage mice showed that T60 SOD1-Thy1-EphA4 mice still had a 30% reduction of EphA4 levels compared to T60 SOD1-EphA4 control mice (Fig. 5E). We found no differences in body weight in T60 SOD1-Thy1-EphA4 mice compared to controls (Supplementary Data, Fig. S2C).

## Discussion

Our results show that 50% or more than 85% ubiquitous or neuronal EphA4 knockdown at a pre-symptomatic phase of the disease, does not delay disease onset or increase survival. Since we previously identified a beneficial effect of reducing EphA4 from developmental stages, this suggests that a crucial time window may exist for a potential EphA4-based intervention in ALS.

In the previous study, we analysed a constitutive EphA4 knockout mouse resulting in EphA4 reduction from developmental stages onwards. In our current work, we have reduced EphA4 levels in SOD1^G93A^ mice ubiquitously or specifically in neurons from the age of 60 days. At this time-point, mice are pre-symptomatic and motor neurons are still preserved. Extensive work on the SOD1^G93A^ model has revealed very early pathological events that occur several weeks prior to the motor symptom onset and motor neuron death. Between postnatal day (P) 45 and P80, before any reduction in the numbers of axons exciting the spinal cord occurs, synapses between fast-fatigable and fast-resistant motor neurons and muscle fibres are already lost^24,25^. Synapses from slow motor neurons are spared until the end of the disease^24,25^. Compensatory mechanisms also play a role in this process, since slow muscle fibres-innervating axons, can re-sprout and re-innervate denervated muscle fibres^25^. We hypothesized that decreasing EphA4 expression from P60 could improve muscle re-innervation and neuronal survival, but we did not observe any delay in disease onset or an extended survival. Despite the fact that EphA4 knockdown occurred very fast after Tamoxifen administration, this time-point may have already been too late to result in a beneficial effect. Supressing EphA4 expression in the SOD1^G93A^ mouse at 50 days of age with ASOs delivered by intracerebroventricular injection did not improve disease parameters either^20^. Reducing EphA4 levels at P50 or P60 can be the right timing to promote re-sprouting of the remaining axons, but too late to avoid the most vulnerable axons to denervate. Another genetic approach in which EphA4 was specifically knocked down from a postnatal age of 5 days in ChAT-expressing neurons, delayed disease onset without altering survival^21^. However, such an early treatment would be complex to perform in patients.

Besides the fact that a therapeutic time window may exist for EphA4 as discussed earlier, other factors that might limit the efficiency of the approach need to be considered as well. Our approaches here are based on a specific genetic knockdown of EphA4, which did not show any benefit for disease progression in an ALS mouse model. EphA4 is one of the 14 Eph receptors of the Eph-ephrin system. Potentially, more than one Eph receptor can modify disease expression in ALS, and therefore targeting multiple Eph receptors may be more effective. In support of this, targeting rho-associated kinase, which is a common downstream effector of Eph receptors among other signalling pathways, improves both disease onset and survival in the SOD1^G93A^ mouse^26,27^. Moreover, when Eph receptors bind an ephrin ligand, bidirectional signalling occurs, both in the cell that expresses the receptor and in the cell that bares the ligand^11^. Interestingly, approaches that tried to modulate ligand binding properties of EphA4 disturbing its signalling, either by using an EphA4 agonist or a fusion protein Fc-EphA4, could extend survival or motor performance in ALS^21,22^. Whereas the first one would activate EphA4 and induce internalization of it, and compete for EphA4 binding to its other ligands and induce reverse signalling, the second one is an antagonist that binds EphA4 ligands and also blocks their signalling. In addition, treatment with an EphA4 blocking peptide also improved onset and survival in a rat model for ALS^10^. Since both an EphA4 agonist and an EphA4-ligand antagonist approach extends survival in rodent models for ALS, further in depth study to unravel the mechanisms of action is clearly required. Since binding promiscuity exists between Eph receptors and ligands, our genetic reduction of EphA4 could have been compensated by other Eph receptors present at the cell membrane, and therefore the signalling mediated by EphA4 ligands may not have been impaired. In summary, it may be that other Eph receptors and/or EphA4 ligands could be involved in the pathophysiology of ALS. We have recently shown that a reduction of ephrin-A5 levels to 50% does not improve but accelerates disease progression in the SOD1^G93A^ mouse model^28^. Whether this effect is caused by inhibitory interactions in *cis* with EphA4 expressed at the same membrane, or by interactions with other Eph receptors, is still unknown. A combined strategy that targets the binding between more than one Eph receptor to the respective ligands has been suggested as a therapy in cancer, and a pan-Eph compound that inhibits binding of Ephs to ephrins has already been synthesised and has given positive results when tested in models of glioblastoma^29^. It would be interesting to study the relevance and safety of such an approach for ALS.

EphA4 expression levels correlate with disease onset and survival in sporadic ALS patients^10,20^. A limitation of our study is that we only determined the effect of lowering EphA4 levels in the SOD1^G93A^ mouse model. Future studies need to address the effect of lowering EphA4 levels in other ALS models and determine whether such strategy would modify disease mechanisms induced by other ALS-causing mutations.

Overall, our findings suggest that a specific EphA4 knockdown in adulthood may have a limited therapeutic potential for ALS. Further work needs to be performed to determine whether a broader strategy that takes into account other Eph receptors or ephrins might be of interest and whether such an approach could have a risk of inducing unwanted toxic effects.

## Materials and Methods

### Animals

Different mouse strains were obtained at the Jackson Laboratory (Ben Harbor, ME): the human mutant SOD1 overexpressing mouse (B6.SOD1^G93A^; B6.Cg-Tg(SOD1*G93A)1Gur/J; stock number 004435), an EphA4 floxed mutant mouse that has loxP sites flanking the exon 3 of the *Epha4* gene (EphA4^tm1.1Bzh^; Epha4tm1.1Bzh/J; stock number 012916), the CAG-CreER^TM^ mouse (B6.Cg-Tg(CAG-cre/Esr1*)5Amc/J; stock number 004682), and the Thy1-CreER^T2^ mouse (SLICK-H; Tg(Thy1-cre/ERT2,-EYFP)HGfng/PyngJ; stock number 012708). All animals were housed under standard conditions according to the guidelines of the University of Leuven (KU Leuven), with a 12h light-dark cycle and with access to food and water *ad libitum* as previously described^28^. Mice were maintained in a C57BL/6J genetic background, and were bred between them to obtain the experimental groups: EphA4^F/+^ (EphA4^het^), CAG-CreER^TM^ EphA4^F/+^ (CAG-EphA4^het^), EphA4^F/F^ (EphA4), CAG-CreER^TM^ EphA4^F/F^ (CAG-EphA4), Thy1-CreER^T2^ EphA4^F/F^ (Thy1-EphA4), SOD1^G93A^ EphA4^F/+^ (SOD1-EphA4^het^), SOD1^G93A^ CAG-CreER^TM^ EphA4^F/+^ (SOD1-CAG-EphA4^het^), SOD1^G93A^ EphA4^F/F^ (SOD1-EphA4), SOD1^G93A^ CAG-CreER^TM^ EphA4^F/F^ (SOD1-CAG-EphA4), SOD1^G93A^ Thy1-CreER^T2^ EphA4^F/F^ (SOD1-Thy1-EphA4). For the mice carrying the SOD1^G93A^ overexpression, only those, whose father had a lifespan shorter than 165 days, were used for breeding and for experimental purposes as previously described^28^. Both males and females were used in every experiment. In order to knock down EphA4, Tamoxifen (Sigma, 200 mg/kg/day) was administered by oral gavage during four consecutive days. All animal experiments were carried out in accordance with the U.K. Animals (Scientific Procedures) Act, 1986 and associated guidelines, EU Directive 2010/63/EU for animal experiments, and all animal experiments were approved by the local ethical committee of the KU Leuven (P229/2013 and P229/2017) and conducted in a blinded manner as previously described^28^.

### Disease phenotype monitoring

Disease phenotype monitoring was performed according to guidelines^30^, and as previously described^28^. Briefly, motor coordination was assessed with the hanging wire test, which records the ability of mice to hang upside down from an elevated grid. The latency to fall was recorded with a cut-off of 60 s. Experimental ALS mice were trained at the age of 50 days during three consecutive days, and next, weight and hanging wire performance were assessed three times every week until mice reached an end stage point of the disease. ALS mice were considered to have reached this end stage point of the disease when, due to paralysis and muscle wasting, their righting reflex took longer than 10 s. At this time-point mice were euthanized according to human end-points, and the mouse age was annotated for survival plots. Disease onset was determined as the age at which mice could no longer perform the maximum score of 60 s in the hanging wire test, and disease duration was calculated as the time between disease onset and mouse survival.

### RNA extraction and qPCR

Lumbar spinal cords were dissected from mice euthanized with cervical dislocation. Total RNA was extracted with TRIzol (ThermoFischer Scientific) and precipitated with isopropanol, as indicated by the manufacturer. cDNA and qPCRs were performed as previously described^28^. Briefly, up to 1 µg of RNA was used to prepare cDNA with the SuperScript III First-Strand Synthesis System (ThermoFischer Scientific) and quantitative PCRs were performed with the StepOnePlus (Life Technologies), the TaqMan Fast Universal PCR Master Mix 2X (Life Technologies) and the following Taqman assays (IDT): *Epha4* (Mm.PT.58.13545379), *Polr2a* (Mm.PT.58.13811327) and *Gapdh* (Mm.PT.39a.1). Relative gene expression was analysed with the Qbase+ software (Biogazelle).

### Protein lysis and western blot

Lumbar spinal cords were dissected from mice euthanized with cervical dislocation. Tissue homogenization was performed as previously described^28^. Briefly, tissue was homogenized in RIPA buffer (Sigma-Aldrich) supplemented with Complete EDTA-free Cocktail (Roche), phosSTOP (Roche) and PMSF (ThermoFischer Scientific), by means of mechanical disruption using Lysing Matrix D beads (MP Biomedicals) and a MagNa Lyser oscillator (Roche) at 6 500 rpm for 30 s thrice with 1 min interval on ice. Next, samples were centrifuged at 14 000 rpm for 15 min at 4 °C and supernatants were collected. Protein concentration was determined with the BCA Kit (ThermoFischer Scientific) according to the manufacturer’s instructions. An amount of 15 µg of protein lysate was mixed with Pierce Lane Marker Reducing Sample Buffer (ThermoFischer Scientific) and heated during 5 min at 95 °C. Samples were resolved in a 4–20% mini-PROTEAN TGX gel (Biorad) and transferred to a polyvinylidene difluoride (PVDF) membrane (Millipore) by a semi-dry transfer apparatus (Bio-Rad) at 180 mA during 1 h 45 min. The membranes were then blocked with 5% milk in TBST (10 mM Tris-HCl (pH 7.5), 150 mM NaCl and 1% Tween-20) for 1 h at room temperature (RT). Membranes were incubated with the following primary antibodies diluted in TBST containing 1% bovine serum albumin (BSA; ThermoFischer Scientific): EphA4 (1: 1000; overnight at 4 °C; Invitrogen, 37–1600), β-actin (1:5 000; 15 min at RT; Sigma, A5441), α-tubulin (1: 10 000; 10 min at RT; Sigma, T6199) and GAPDH (1:10 000; 1 h at RT; Ambion, AM4300). Membranes were washed with TBST and incubated at RT during 1 h with goat anti-mouse HRP-conjugated immunoglobulins (DAKO; P044701-2) diluted 1:5 000 in TBST containing 5% milk. Finally, membranes were developed with enhanced chemiluminescence (ECL) western blotting substrate (ThermoFischer Scientific), scanned using a LAS4000 Biomolecular imager (GE Healthcare) and analysed with ImageQuant TL version 7.0 software (GE Healthcare).

### RNAscope *in situ* hybridization

Samples preparation and RNAscope *in situ* hybridization was performed as previously described^28^. Briefly, 20 µm-cryosections were obtained from fixed-frozen lumbar spinal cord samples with a CryoStar NX70 Cryostat (ThermoFischer Scientific). RNAscope was next performed with the RNAscope Multiplex Fluorescent Reagent Kit v2 (ACD Diagnostics) as indicated by the manufacturer. In brief, slides underwent an antigen retrieval step of 5 min at 98–104 °C with a Braun Multiquick FS-3000 Steamer (Braun) and a Protease III incubation step of 30 min at 40 °C in a HybEZ™ Oven (ACD Diagnostics). Sections were incubated for 2 h at 40 °C with RNAscope probes against *Epha4* (RNAscope Probe - Mm-Epha4-C1; ACD Diagnostics) and *Synaptophysin* (*Syp*; 1:100; RNAscope Probe - Mm-Syp-C3; ACD Diagnostics). Signal amplification was performed as stated in the manufacturer’s instructions with TSA Plus Cyanine 3 (1:500; Perkin Elmer) and TSA Plus Cyanine 5 (1:10 000; Perkin Elmer). Nuclear counterstain was performed with Hoechst 33342 (5 µg/ml; Sigma) and slides were finally mounted with ProLong Gold antifade reagent (Life Technologies). Image acquisition of the ventral horns of every spinal cord section was performed with a Leica TCS SP8 confocal laser scanning microscope (Leica Microsystems Heidelberg GmbH, Manheim, Germany) with an HC PL APO CS2 20x/0.75 dry lens and a pinhole of 0.5 Airy Units. Sum projection of 2 Z-stacks separated 2 µm from each other was done with the freeware ImageJ v1.51 u by Wayne Rasband (National Institutes of Health, Bethesda, MD, USA) and images were next automatically quantified with the NIS-Elements Microscope Imaging Software (Nikon). *Epha4* dot density was quantified within the neuronal fraction and also within the glial cells as previously described^28^. *Syp* expression was used to designate neurons. All detected nuclei that did not colocalize with *Syp*-positive cells were considered as glial nuclei, and a small perimeter (NIS-Elements thickening command, three iterations) around each nucleus was also selected to detect part of the cytoplasm of these cells. Glial nuclei with the small perimeter around them were considered as glial cells.

### Statistics

Graphpad prism 7.01 software (Graphad Software) was used for statistical analysis. Different tests were performed to determine statistical significance as it is indicated in the figure legends. Survival and disease onset were analysed using the Log-rank test as previously described^28^. Significance level was considered for p values lower than 0.05. Graphs represent data with SEM values.

Based on previous experiments in SOD1^G93A^ mice, we estimated that a sample size of 32 mice in each group would provide 80% power at an α = 0.05 to detect an extended survival of 11 days, as previously described and as previously found for EphA4^+/−^ SOD1^G93A^ mice^10,28^. We discontinued the experiments before reaching the required sample size for ethical reasons, since the lack of any difference after including more than half of the required animals.

## Supplementary information

Supplementary Figures

## Data Availability

No datasets were generated or analysed during the current study.
