# Supplementary material for: Reducing EphA4 before disease onset does not affect survival in a mouse model of Amyotrophic Lateral Sclerosis
Source: Sci Rep. 2019 Oct 1;9:14112. doi: 10.1038/s41598-019-50615-0 (PMC6773754; doi:10.1038/s41598-019-50615-0)

# Reducing EphA4 before disease onset does not affect survival in a mouse model of Amyotrophic Lateral Sclerosis

Laura Rué^1,2^, Mieke Timmers^1,2^, Annette Lenaerts^1,2^, Silke Smolders^1,2^, Lindsay Poppe^1,2^, Antina de Boer^1,2^, Ludo Van Den Bosch^1,2^, Philip Van Damme^1,2,3^, Wim Robberecht^1,3^, Robin Lemmens^1,2,3^

^1^ KU Leuven – University of Leuven, Department of Neurosciences, Experimental Neurology and Leuven Brain Institute (LBI), Leuven, Belgium

^2^ VIB, Center for Brain & Disease Research, Laboratory of Neurobiology, Leuven, Belgium

^3^ University Hospitals Leuven, Department of Neurology, Leuven, Belgium

**Supplementary material**


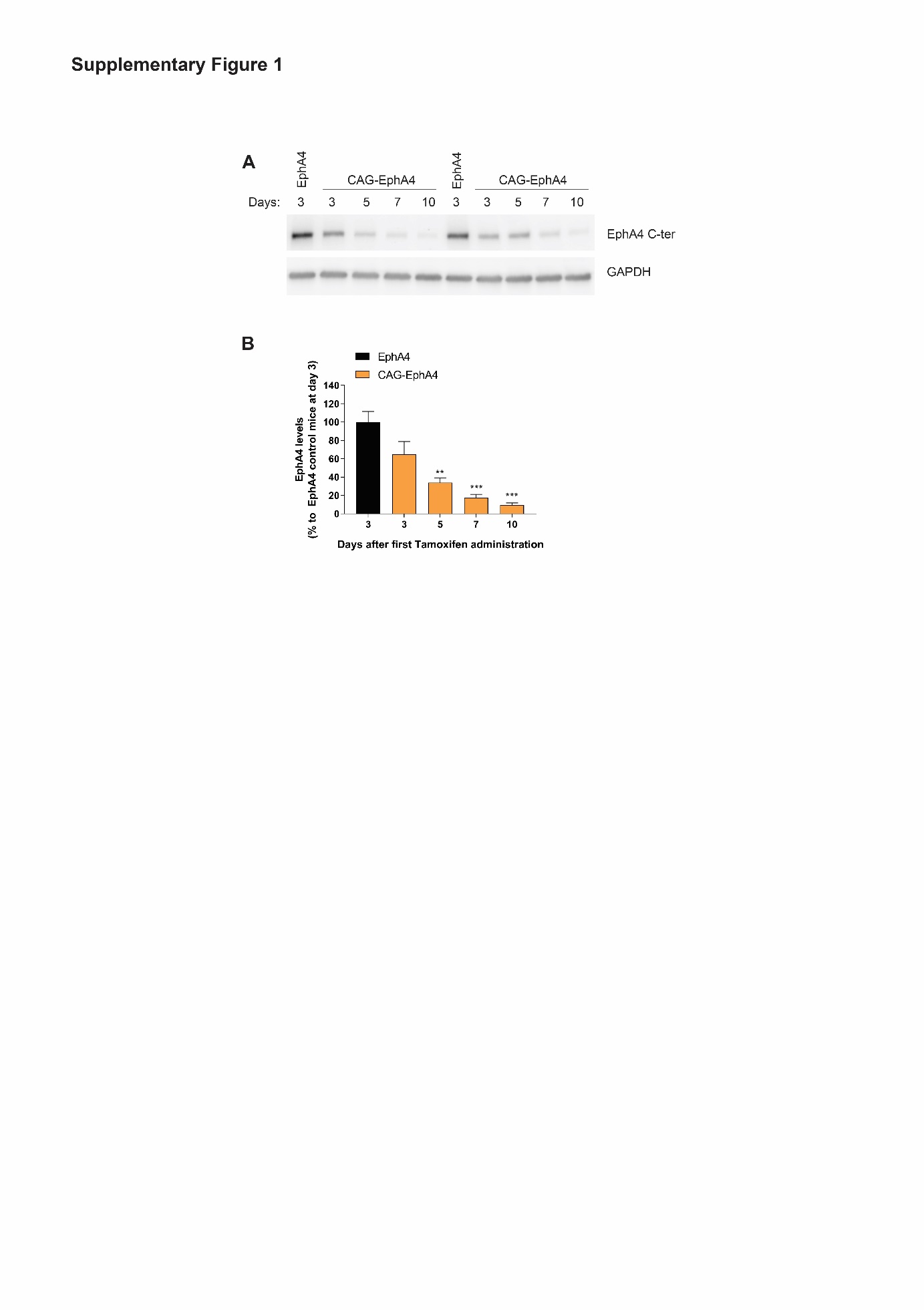


Supplementary Figure S1. Ubiquitous EphA4 knockdown is efficient and occurs fast after Tamoxifen administration. (A, B) Time-course of EphA4 knockdown was assessed at 3, 5, 7 and 10 days after the first Tamoxifen administration dose, which consisted in total in 4 doses administered during 4 consecutive days. (A) A representative immunoblot is shown, where EphA4 C-terminal (EphA4 C-ter) antibody was used to quantify EphA4 levels and GAPDH was used as a loading control. Full-length western blot can be found in at the end of this document. (B) Immunoblots were quantified and analysed with a one-way ANOVA, followed by a Dunnett's multiple comparisons test: ** *P* < 0.01, *** *P* < 0.001 as compared to EphA4 mice 3 days after the first Tamoxifen dose.


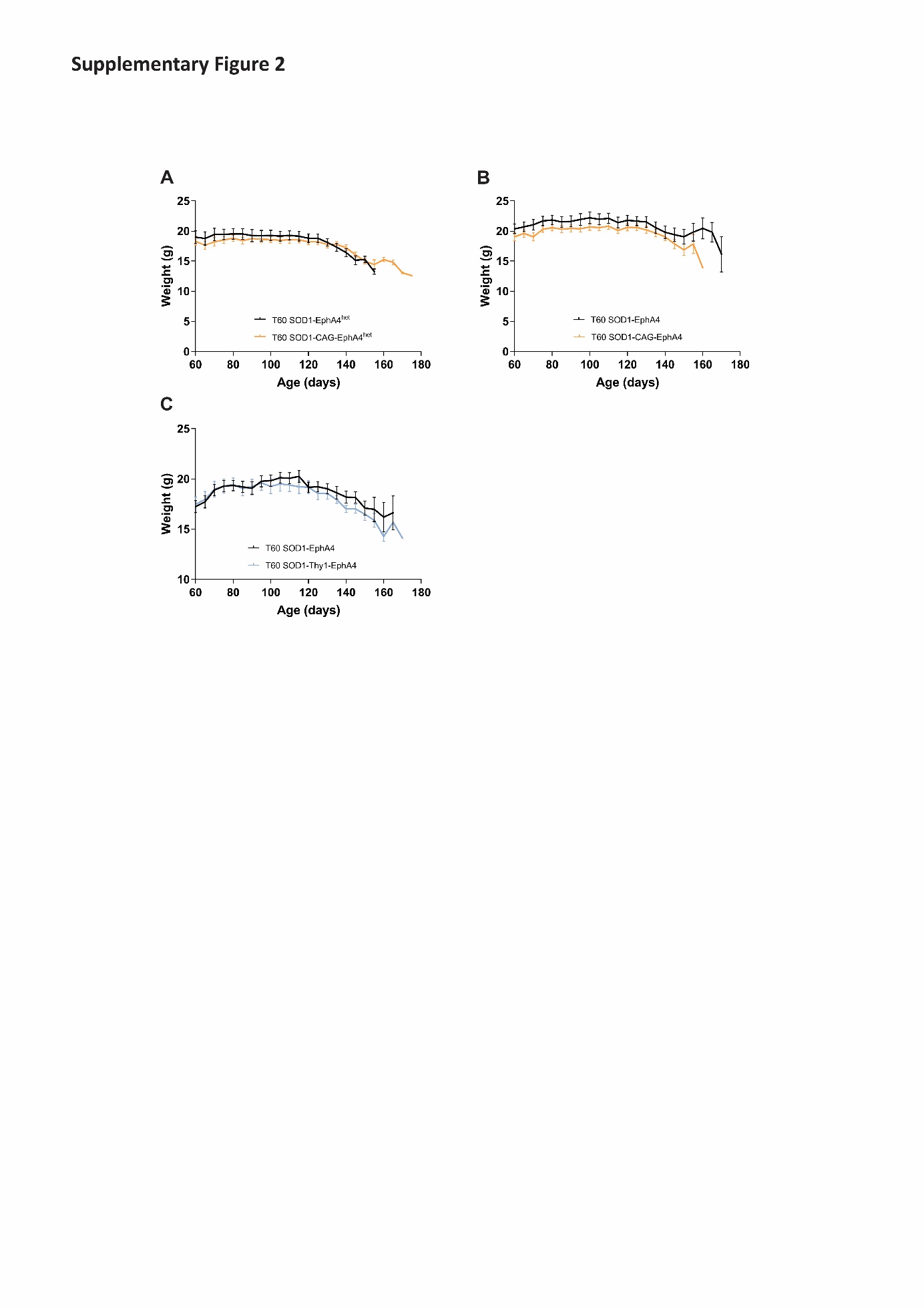


Supplementary Figure S2. Weight is not altered in mice with reduced EphA4 levels. (A-C) Weight was assessed regularly from the age of 60 until mice reached an end-stage point of the disease in (A) T60 SOD1-CAG-EphA4^het^ mice, (B) T60 SOD1-CAG-EphA4 mice, and (C) T60 SOD1-Thy1-EphA4 mice and their respective littermate controls. Data represents mean ± SEM in grams (g).


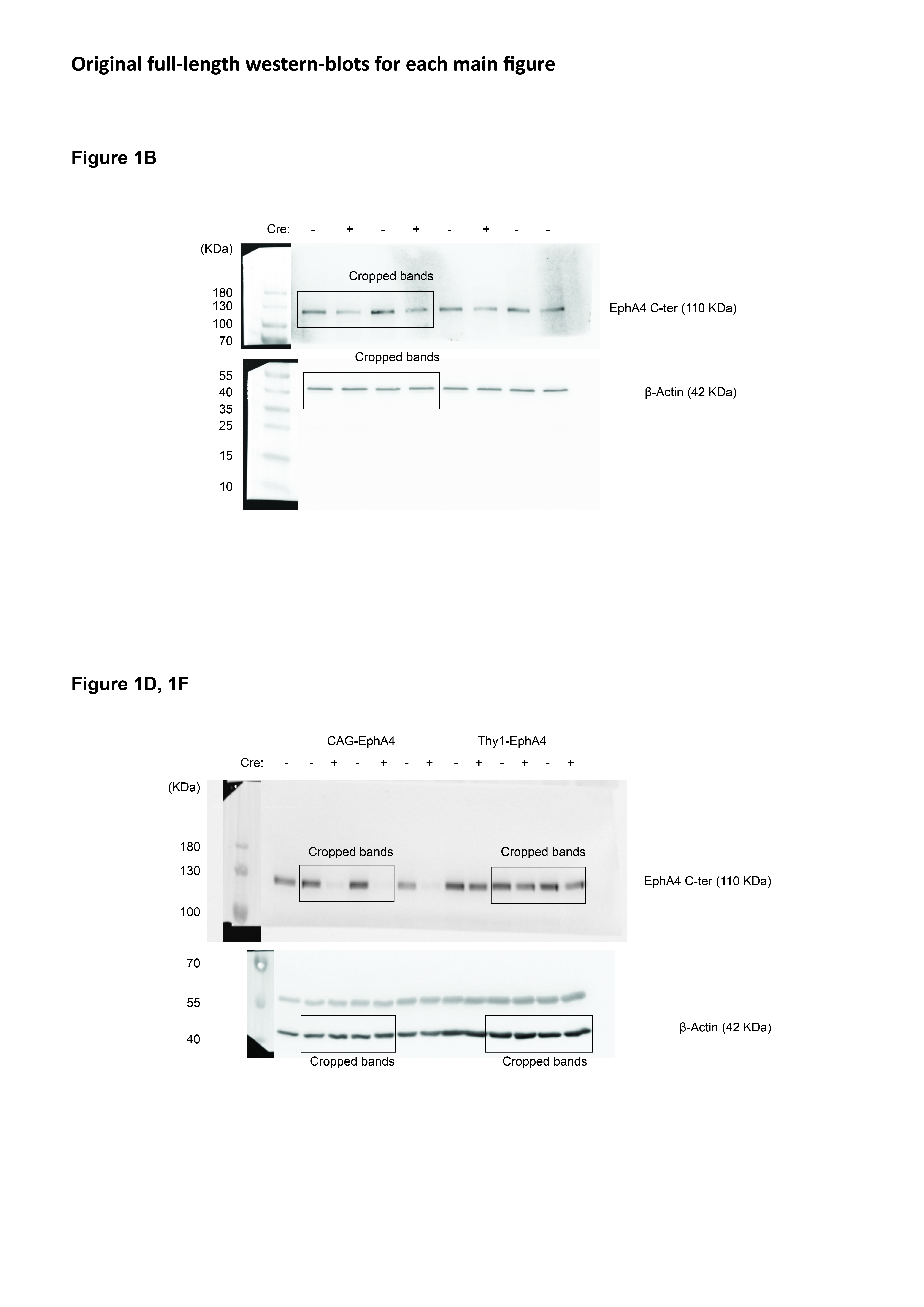

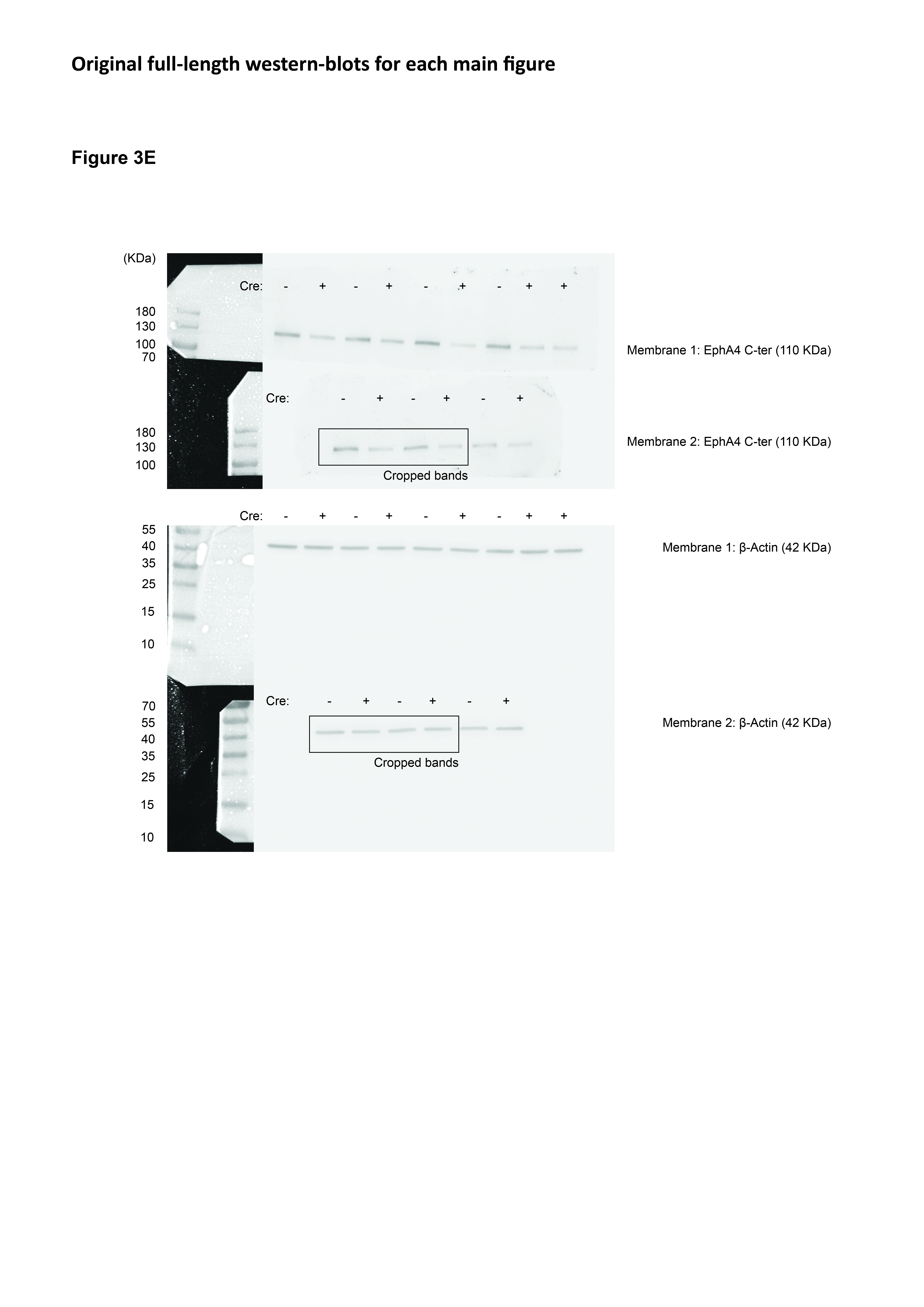

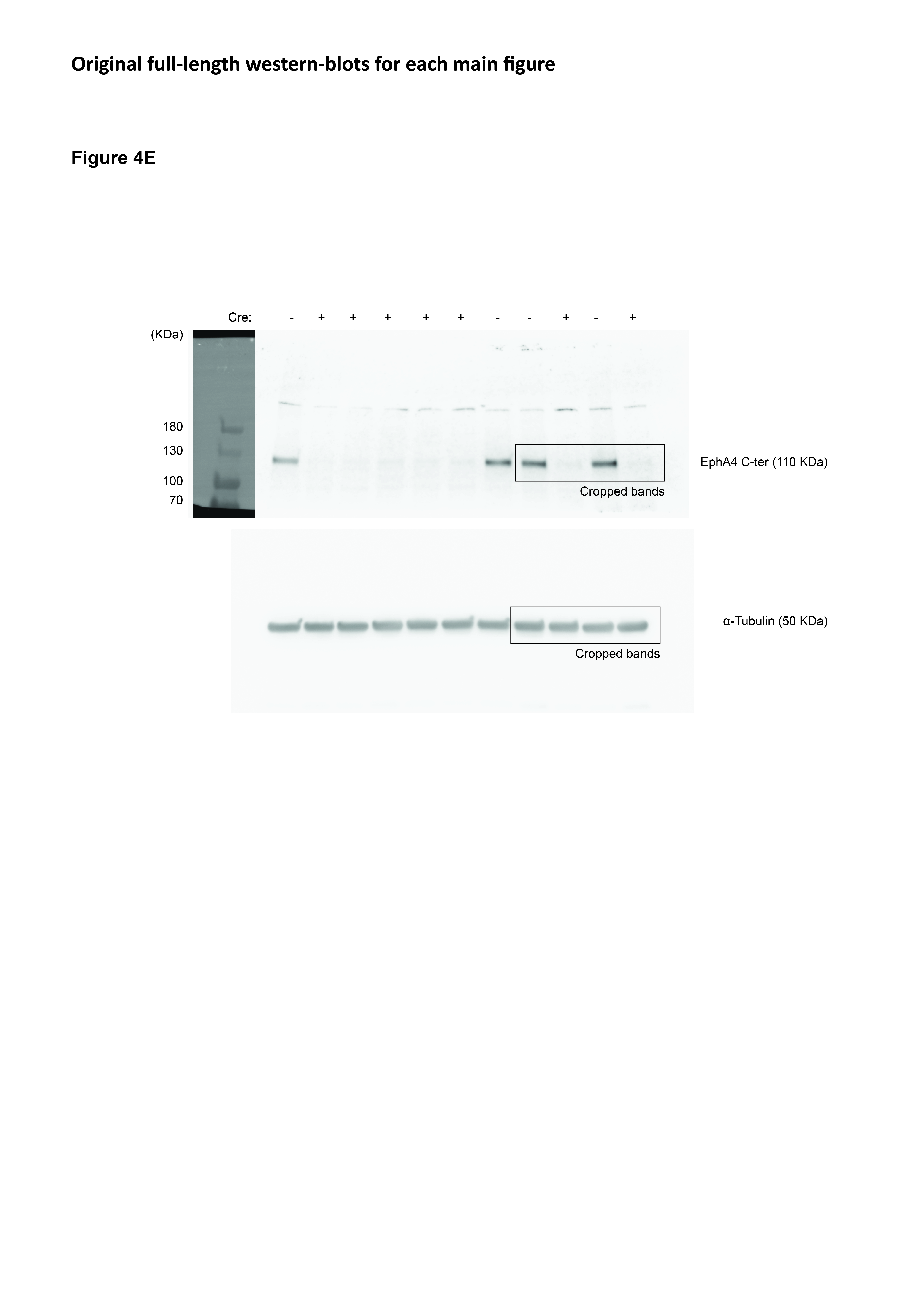

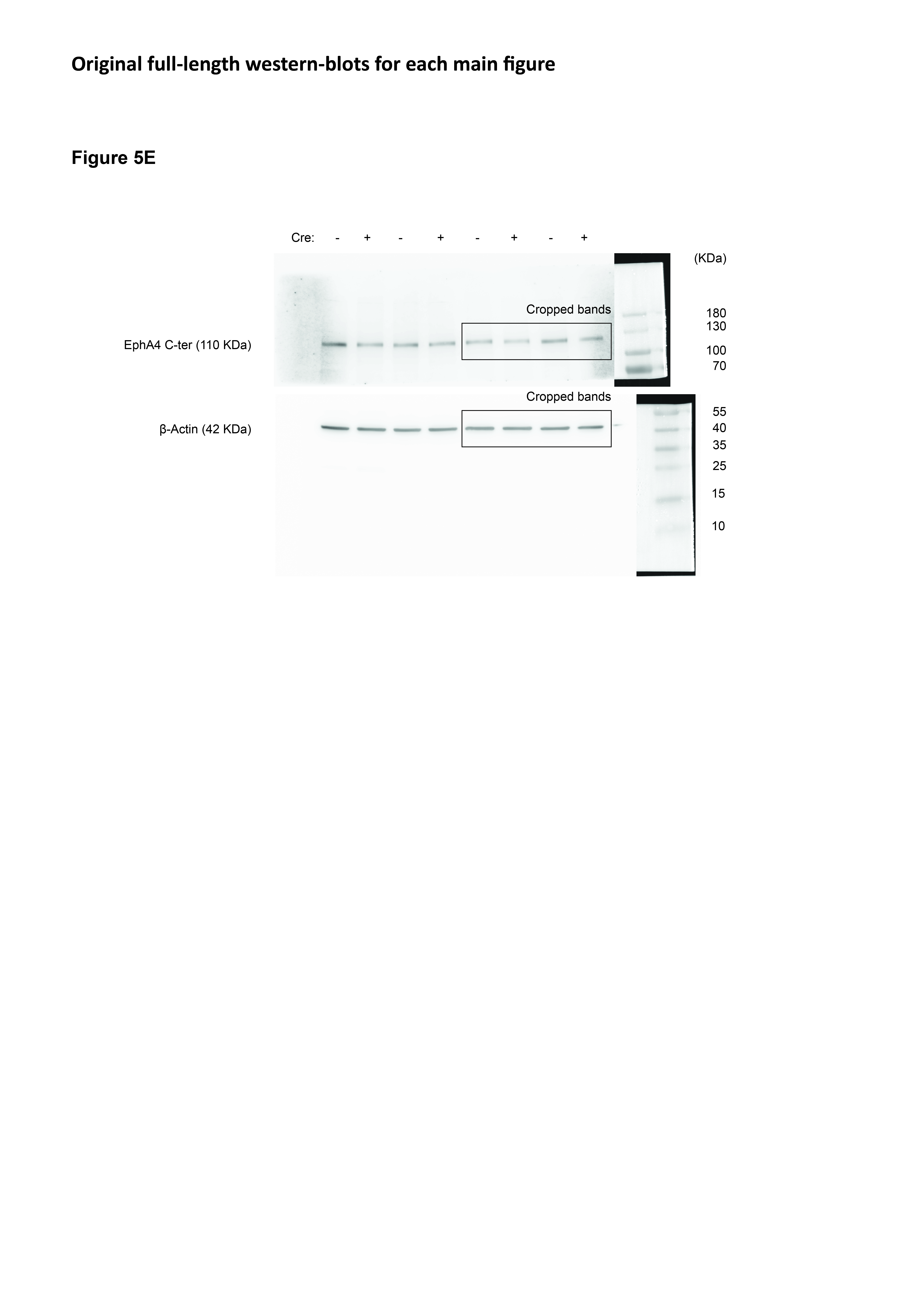

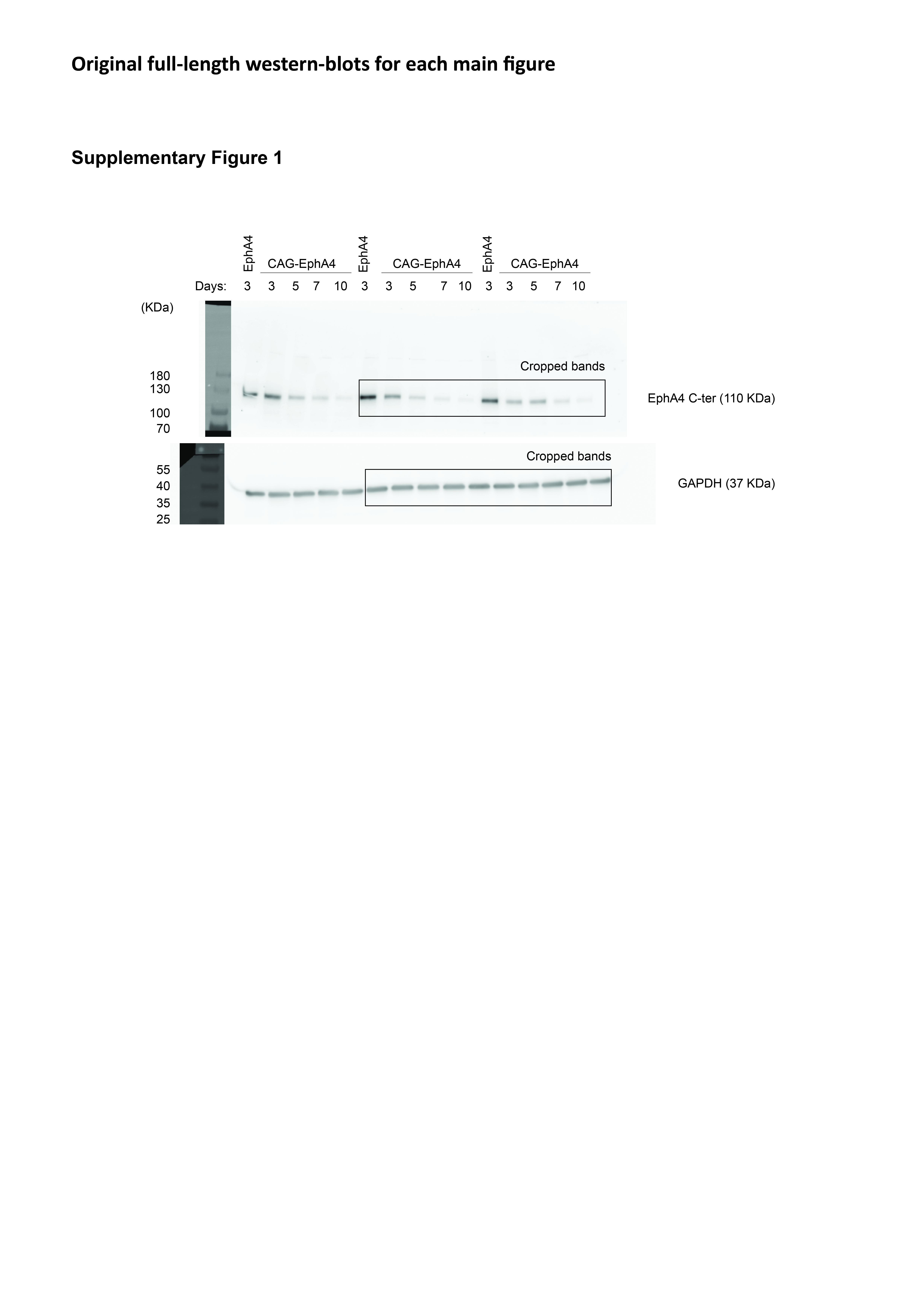

Supplement: Supplementary file 1 — Supplementary Figures [file 41598_2019_50615_MOESM1_ESM.docx]
